# DNA methylation regulates mouse cardiac myofibril gene expression during heart development

**DOI:** 10.1186/s12929-015-0203-6

**Published:** 2015-10-17

**Authors:** Yang Xu, Lingjuan Liu, Bo Pan, Jing Zhu, Changlong Nan, Xupei Huang, Jie Tian

**Affiliations:** Department of Cardiology, Heart Centre, Children’s Hospital of Chongqing Medical University, 136 Zhongshan 2nd Road, Yu Zhong District, Chongqing, 400014 P.R. of China; Ministry of Education Key Laboratory of Child Development and Disorders; Key Laboratory of Pediatrics in Chongqing, CSTC2009CA5002; Chongqing International Science and Technology Cooperation Center for Child Development and Disorders, Chongqing, P.R. of China; Department of Biomedical Science, Charlie E. Schmidt College of Medicine, Florida Atlantic University, 777 Glades Road, Boca Raton, FL 33431 USA

**Keywords:** Troponin I, DNA methylation, 5-Azacytidine, Epigenetic regulation

## Abstract

**Background:**

It is well known that epigenetic modifications play an important role in controlling the regulation of gene expression during the development. Our previous studies have demonstrated that the expression of fetal troponin I gene (also called slow skeletal troponin I, ssTnI) is predominated in the fetal stage, reduced after birth and disappeared in the adulthood. The mechanism underlying the developmentally related ssTnI gene regulation is not clear. In this study, we have explored the epigenetic role of DNA methylation in the regulation of ssTnI expression in the heart during the development.

**Results:**

The DNA methylation levels of CpG island and CpG dinucleotides region were detected using methylation specific PCR (MSP) and bisulfite sequence PCR (BSP) in 2000 bp upstream and 100 bp upstream of ssTnI gene promoter. Real time RT-PCR and Western blot were used to detect ssTnI mRNA and protein expression levels. We found that DNA methylation levels of the CpG dinucleotides region in ssTnI gene promoter were increased with the development, corresponding to a decreased expression of ssTnI gene in mouse heart. However the DNA methylation levels of CpG islands in this gene were not changed during the development. Application of a methylation inhibitor, 5-Azacytidine, in cultured myocardial cells partially prevented the decline of ssTnI expression.

**Conclusion:**

Our results indicate that DNA methylation, as an epigenetic intervention, plays a role in the regulation of the fetal TnI gene expression in the heat during the development.

## Background

Sarcomere protein, troponin, is located on the thin filament of myocardial cells and plays an essential role in regulating Ca^2+^-activated tension of striated muscles. Troponin component contains three isoforms: troponin T (TnT), binding to tropomyosion forming troponin-tropomyosion complex, troponin C (TnC), binding to Ca^2+^ to produce a conformational change in TnT, and troponin I (TnI), an inhibitory subunit binding to actin-tropomyosion and regulating muscles contraction [1]. There are at least two developmentally regulated TnI isoforms in the heart: the slow skeletal TnI (ssTnI) that is expressed in the fetal heart and the cardiac TnI (cTnI) that is predominately expressed in adult hearts [1–3]. TnI isoform switching is common in animals and human, and it is a good model to investigate the regulation of cardiac proteins in the development [4].

It is clinically important to clarify the regulation of TnI expression, because many cardiomyopathy and heart diseases are associated with abnormal TnI protein expression leading to diastolic dysfunction and heart failure [5–7]. In our previous studies, we have demonstrated that ssTnI expression in heart is partially regulated by thyroid hormone during the heart development [8, 9]. And we have also cloned mouse ssTnI gene with upstream promoters and revealed several regions and domains on the promoters critically to ssTnI gene expression, such as TnI slow upstream regulatory elements (SURE), Yin Yang 1 factor (YY1), proximal 300 bp upstream region and the first intron of ssTnI gene [10–13]. However, the mechanisms of ssTnI down-regulation and finally shut down in the heart after birth is still not clear.

DNA methylation is one of epigenetic modifications and many studies have showed that DNA methylation plays an important role in gene expression, genomic imprinting, X-chromosome inactivation and chromatin structure changes [14–16]. DNA methylation occurs on position 5 of cytosine by the covalent modification of a methyl group, creating 5-methylcytosine, which is preferentially found in CpG dinucleotides [17]. The “CpG” is shorthand for “-C-phosphate-G-”, which is cytosine and guanine separated by only one phosphate. CpG island is a region with high frequency of CpG sites (GC percentage is greater than 50 % in a region over 200 bp). In addition, some CpG sites that are in a short distance from CpG island are called CpG island sites (or CpG shore), which also play an important role in regulating gene expression [18]. The methylation of CpG dinucleotides in promoter of genes always leads to a transcriptional inactivation. Some studies have shown that different levels or patterns of cytosine methylation are found in various tissues or in different functional regions of the same tissue [19–21]. Our previous study has shown that epigenetic modification may regulate ssTnI expression during heart development, especially through histone acetylation and histone methylation [22]. However, the role of DNA methylation in the regulation of ssTnI gene expression is still unknown. In the present study, we have measured the levels of DNA methylation in critical domains of ssTnI genes to determine the role of epigenetic regulation on this gene expression. Furthermore, a DNA methyltransferase inhibitor, 5-azacytidine has been applied to myocardial cells to confirm the DNA methylation mediated ssTnI gene expression in the heart. Our results indicate that DNA methylation plays an important role in the regulation of ssTnI expression in the heart during the development.

## Methods

### Experimental animals

All animal procedures were approved by the Animal Care and Use Committee at the Chongqing Medical University. Adult wild type KM mice (body weight of 18–22 g) were purchased from Chongqing Medical University Animal Center. Upon arrival, breeder mice were separately housed and acclimated for at least one week before mating began. The mice were maintained on a reverse 12 h light-dark cycle (light 19:00–07:00) and provided with laboratory chow and water ad libitum. Two females were placed with one male for two hours between 08:00 and 10:00. When a vaginal plug was detected after mating period, which was designated as embryonic 0.5 day (E0.5). E14.5, E17.5 pregnant mice, postnatal day 1, day 7, day 14 and adult mice were sacrificed using CO_2_ gas. Embryonic and postnatal heart tissue were collected and frozen at −80 °C until use.

### Culture of mouse myocardial cells

Primary cultures of myocardial cells were performed as described previously [23]. Cardiac myocytes were collected from the mice of postnatal day 14 mice. The cells were randomly divided into two groups, the untreated control group (treated with nothing), 5-azacytidine group (5 μM 5-azacytidine). 5-azacytidine (Sigma, Santa Clara, California, USA) was dissolved in tissue culture medium to make a stock solution and stored in −20 °C. After treated with 5-azacytidine for 24 h, cells were collected and stock at −80 °C for further analyses.

### Real-Time RT-PCR

The real-time PCR was carried out as described previously [10, 11]. The data were analyzed using 2^-[△Ct (target) - △Ct (input)]^ method [24] following the manufacture’s instruction. The primers for PCR amplification: ssTnI forwarding primer: 5′-CTCCACGAGGACTAAACTAGGC-3′, reversing primer: 5′-CTTGGATTTCCTCTCAACTTCC-3′. β-actin forwarding primer: 5′-CACACCCGCCACCAGTTCG-3′, reversing primer : 5′-GTCCTTCTGACCCATTCCCACC-3′.

### Western blot

Western blotting assays for ssTnI were performed as previously describe [8]. An anti-TnI monoclonal antibody (TnI-1) that recognized mouse ssTnI was used at a dilution of 1:10,000. The immune-reactive protein bands were visualized with Chemiluminescent Luminol Reagent (Merck Millipore, USA). After scanning, protein bands were analyzed with Quantity One Version 4.4 software (Bio-Rad, CA, USA).

### Methylation specific PCR (MSP)

Genomic DNA was isolated from ventricular tissue and myocytes cells using a TIANamp Genomic DNA Kit (Tiangen, Beijing, China). DNA methylation of ssTnI promoter CpG islands and CpG dinucleotides region were detected by methylation specific PCR (MSP) using 2 × Power Taq PCR MasterMix (Bioteck, Beijing, China) in a final volume of 25 μl. Bisulfite treatment of unmethylated DNA converted cytosines to uracils at CpG dinucleotides. However, methylated cytosines were not converted. Specific primers were designed to amplify the target regions of interest with unmethylated CpG dinucleotides by detecting uracils and methylated CpG dinucleotides by detecting cytosines. Primer were designed using MethPrimer software (http://www.urogene.org/cgi-bin/methprimer/methprimer.cgi). M pair was indicated methylation of CpG sites within the primer sequences, U pair indicated no methylation, and both pairs indicated a partial methylation: ssTnI CpG island forwarding M primer: 5′-TTGGGGTAGTAGGGTAGAGATATTC-3′, reversing M primer: 5′-TTCTCTTATTCTAAATTCCAACGTC-3′. Forwarding U primer: 5′-TTGGGGTAGTAGGGTAGAGATATTT-3′, reversing U primer: 5′-TTCTCTTATTCTAAATTCCAACATC-3′. ssTnI CpG dinucleotides region forwarding M primer: 5′-ACGGTAGTATATATTTGTTTTGCGA-3′, reversing M primer: 5′-ACTATAAAAACCGTAACCTCCGAC-3′. Forwading U primer: 5′-ATGGTAGTATATATTTGTTTTGTGA-3′, reversing U primer: 5′-AACTATAAAAACCATAACCTCCAAC-3′.

### Bisulfite Sequencing PCR (BSP)

To verify the methylation level of these heart tissues and myocytes cells, bisulfite sequencing PCR (BSP) was used. DNA was first modified by treatment with sodium bisulfite to convert all ‘C’s to uracil residues except 5 mCs. Then bisulfite-modified DNA were amplified by PCR, which performed in a RT-PCR instrument (MJ Mini Personal Thermal Cycler, BIO-RAD) using 2 × Power Taq PCR MasterMix (Bioteck, Beijing, China) under the Touch-down program: 95 °C for 3 mins, followed by 9 cycles of 94 °C for 30 s, 60 °C for 30 s (decrease 1 °C per cycle), 72 °C for 1 min, then followed by 40 cycles of 94 °C for 30 s, 50 °C for 30 s, 72 °C for 1 min, and final extension 72 °C for 10 mins. The BSP primers were designed using MethPrimer: ssTnI CpG island forwarding primer: 5′-TGGGGTTAGAGTGTAAAGTTAATATTG-3′, reversing primer: 5′-TATAACTCCAAACACCCATCTCTCT-3′. ssTnI CpG dinucleotides region forwarding primer: 5′-TTGGTTTTTAAGTTTGTGGTTTATA-3′, reversing primer: 5′-CTAAACTAACCTAAACCTCACCACAA-3′. The resulted PCR product were used to direct sequencing at INVITROGEN (Shanghai, China) to examine bisulfite conversion rate for different time heart tissue [25]. Moreover, the PCR product were recovered by TIANgel Midi Purification Kit (TIANGEN, Beijing, China) after verification in a 2 % agarose gel. Then the purified DNA was ligated into the vector pGM-T by pGM-T Cloning Kit with Competent Cell (TIANGEN, Beijing, China) and transformed into *E. coli* strain TOP10. Sequence determinations were carried out at SANGON (Shanghai, China) [14].

### Statistics

All RT-PCR and Western blot data are showed as mean ± SD. BSP sequences data are analyzed using software from BiQ Analyzer (http://biq-analyzer.bioinf.mpi-inf.mpg.de/) to calculate the level of DNA methylation (methylation level = methylated CpG dinucleotides/total CpG dinucleotides). Statistical analysis was carried out by SPSS 19.0 using ANOVA and Student’s *t*-test to determine statistical significance. The criteria for significance were defined as p <0.05.

## Results

**Fig. 1:**
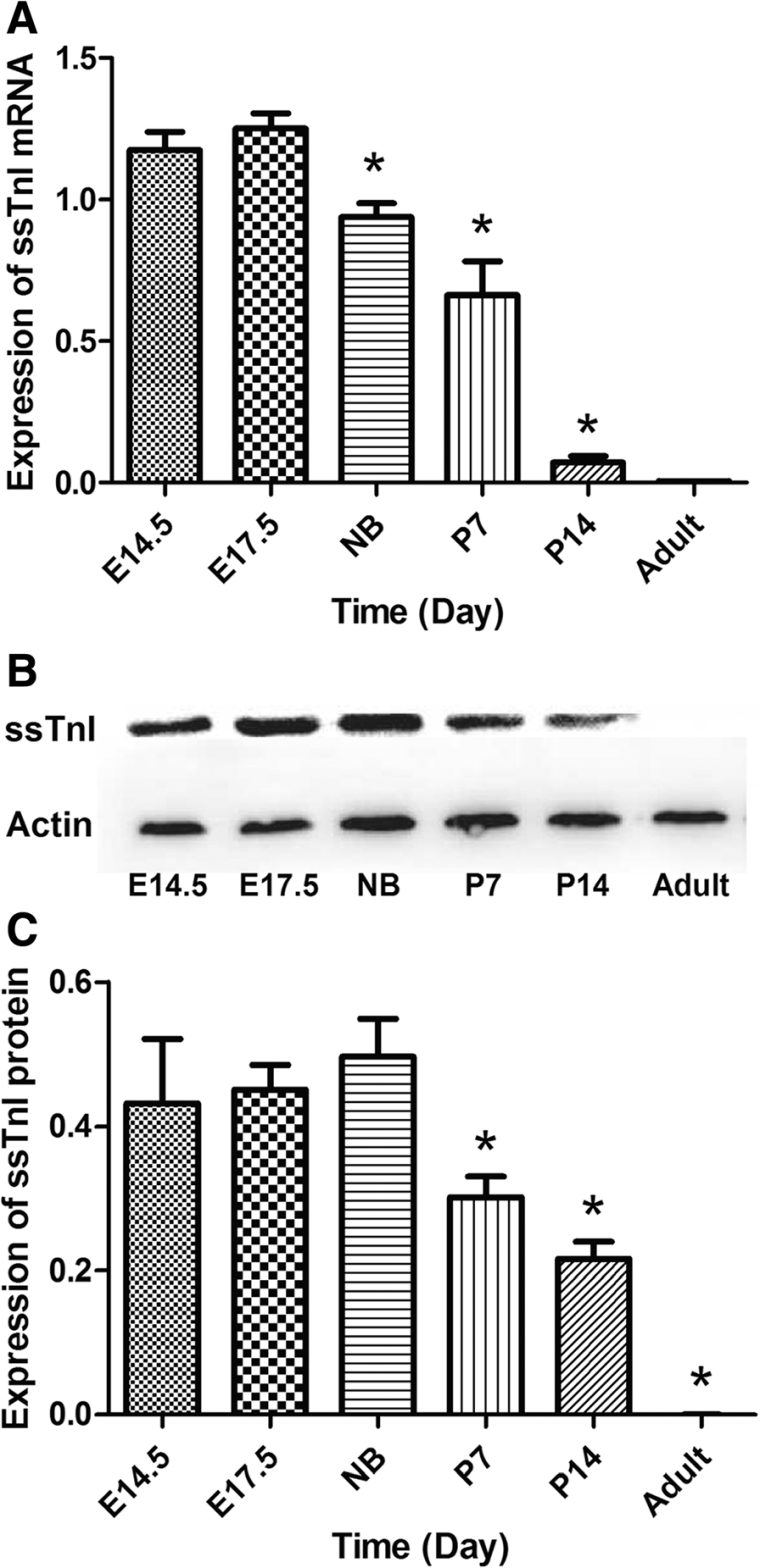
Time course of ssTnI mRNA and protein expression in the heart during development. **a** Expression of ssTnI mRNA is maintained at a high level until newborn. After birth, ssTnI expression in the heart is decreased. **b** Representative immunoblot of ssTnI protein content in mouse heart during development. **c** Summary of the ssTnI protein content in the mouse heart during development. The ssTnI protein expression in the heart is consistent with the mRNA expression pattern observed in the same heart. ANOVA showed a significantly different the expression levels in experimental groups, and *asterisk* indicates *p* < 0.05 in two groups comparison by *t* test between E14.5 group and other groups. E14.5: embryonic day 14.5, E17.5: embryonic day 17.5, NB: newborn, P7: postnatal day 7, P14: postnatal day 14

The time course of the expression of ssTnI mRNA and protein in mouse heart during embryonic day 14.5 to adult is shown in Fig. 1. The highest expression level of ssTnI mRNA was observed in embryonic day 14.5 and 17.5. The expression of ssTnI was declined gradually after birth. In the adult heart, ssTnI expression was barely detected (Fig. 1a). The time course of ssTnI protein levels (Fig. 1b and c) was similar to that of ssTnI mRNA during the heart development, which is consistent with what we reported previously [8].

**Fig. 2:**
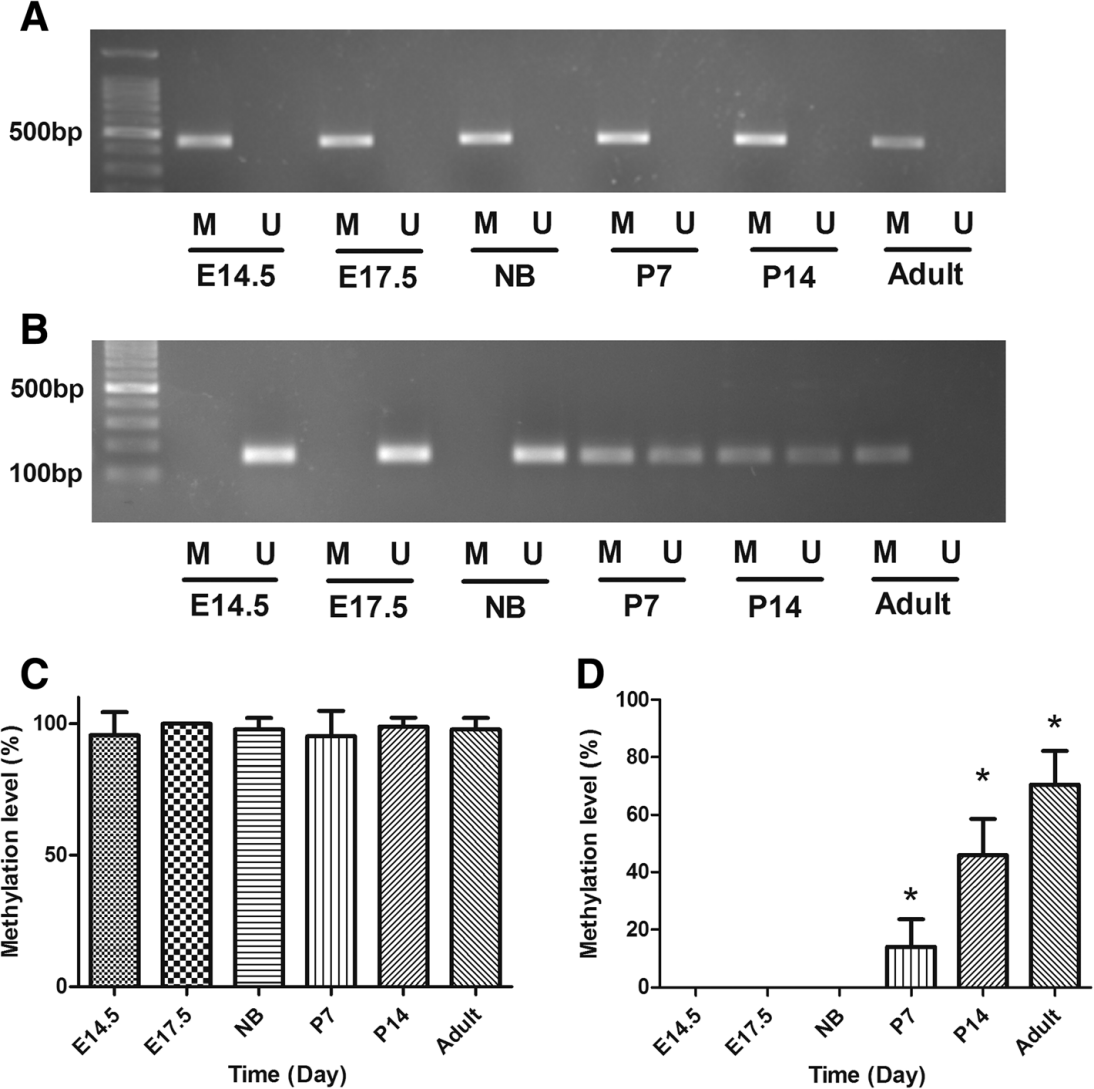
DNA methylation of CpG islands and CpG dinucleotide regions in ssTnI gene promoter. **a** DNA methylation of CpG islands in ssTnI promoter. The CpG island is fully methylated in the cardiac tissues at different time points during development. **b** CpG dinucleotides methylation in -100 bp region away from ssTnI transcription starting site (TSS). In E14.5, E17.5 and NB, the CpG dinucleotides are unmethylated, but in P7, P14, those regions show a partial methylation. In adult, it is fully methylated. M, methylated; U, unmethylated. **c** The ssTnI CpG island methylation levels observed in mouse cardiac tissues during development. Methylation on ssTnI CpG islands is stably maintained in a high level during heart development. **d** CpG dinucleotides methylation levels observed in ssTnI promoter -100 bp. The bisulfite sequence data show that an altered DNA methylation level of CpG site in ssTnI -100 bp region during heart development. ANOVA analyses indicate a significantly different in methylation levels among various experimental groups, and *p < 0.05 indicates a statistical difference in two groups compared using *t* test

The MethPrimer was used to predict CpG islands and CpG dinucleotides regions in ssTnI gene promoter. Only one CpG island was found in the upstream of ssTnI gene, which is about 2000 bp away from the transcription start site (TSS). However, various CpG dinucleotides were found in different domains of the ssTnI gene promoter. One CpG dinucleotides region containing 5 CpG sites was found in about 100 bp from TSS on ssTnI gene. MSP assays were performed to determine DNA methylation on ssTnI gene promoter. The DNA methylation of CpG island in ssTnI was shown in Fig. 2a, indicating that all of CpG islands were fully methylated in all of the time points tested during the heart development. However, the methylation of CpG dinucleotides region was different, which showed that the CpG sites was unmethylated in embryonic day 14.5, 17.5 and newborn, but after birth on postnatal day 7 and 14, methylation occurred on these sites and reached a peak level of methylation in adulthood (Fig. 2b). The gradual methylation of these sites on ssTnI gene shortly after birth is corresponding to a gradual decline of the ssTnI gene expression in the heart during the same time course.

Bisulfite sequence PCR (BSP) assays were carried out to confirm the data obtained from MSP assays. The data from BSP showed that DNA methylation in CpG island of ssTnI gene was not significantly changed during heart development, however, the DNA methylation levels in CpG dinucleotides regions showed that these sites were not methylated during the embryonic stage and even at newborn in the heart and methylation started on these site shortly after the birth, which are consistent with the results from MSP assays.

**Fig. 3:**
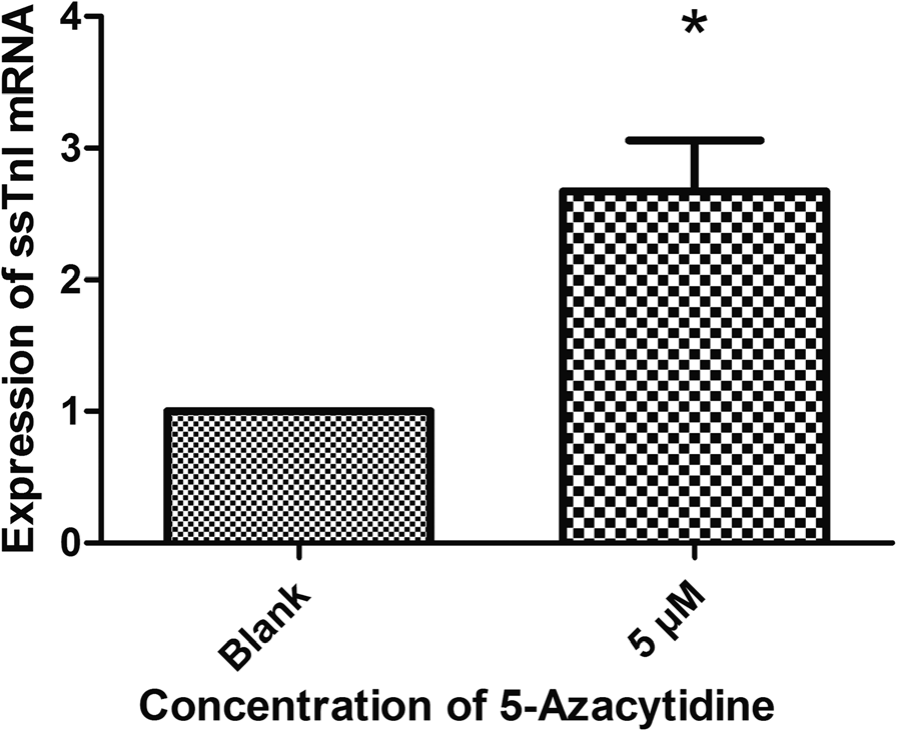
Expression of ssTnI mRNA in myocardial cells with or without treatment of 5-azacytidine. Myocardial cells were isolated from the heart at age of 14 days. One group was treated with 5 μM 5-azacytidine for 24 h, and another group was treated with medium without 5-azacytidine as controls. The quantitative RT-PCR data show a significantly increase of ssTnI mRNA in the cells treated with 5 μM 5-azacytidine. *p < 0.05 indicates a statistical difference in two groups compared using *t* test

**Fig. 4:**
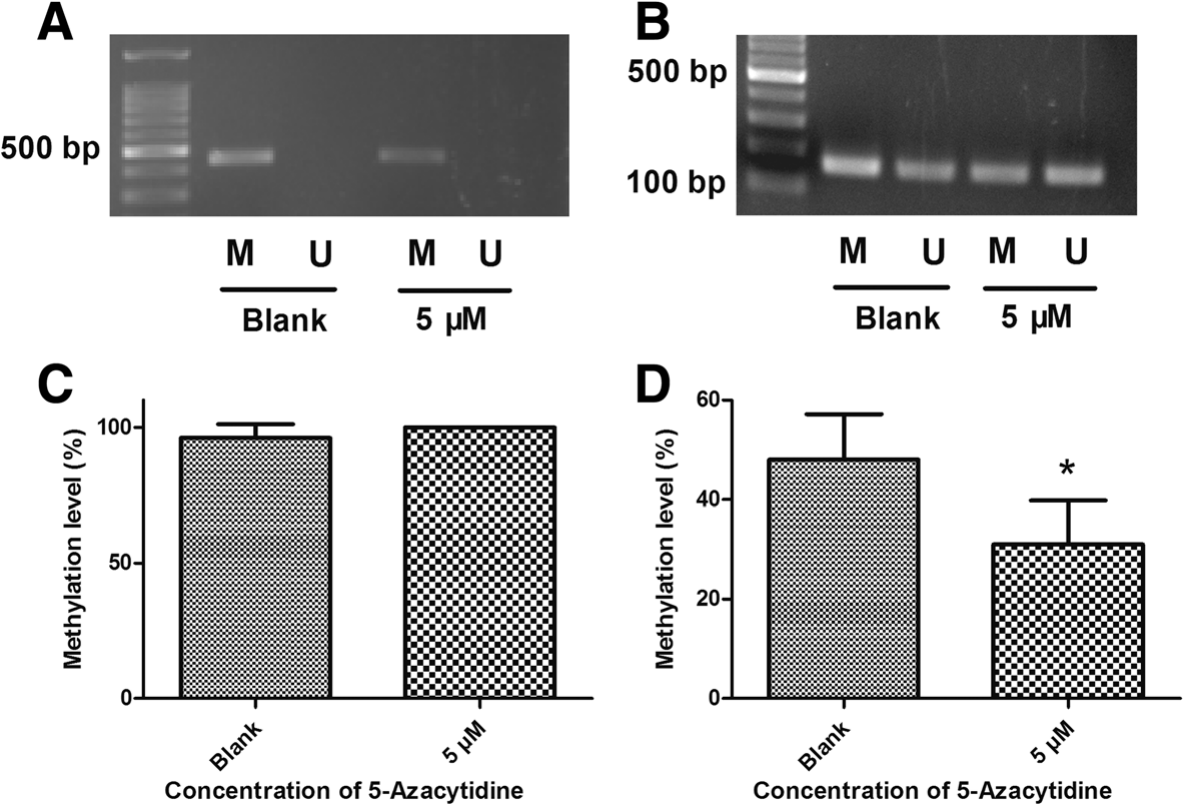
DNA methylation of CpG islands and CpG dinucleotide regions in myocardial cells with or without treatment of 5-azacytidine. Myocardial cells were isolated from 14-day-old mouse hearts. One group was treated with 5-azacytidine and another group was treated with medium without 5-azacytidine as the control. **a** MSP results of CpG island methylation in ssTnI promoter. The CpG islands are methylated both in blank and 5 μM 5-azacytidine treated groups. **b** MSP results of CpG dinucleotide region methylation in ssTnI promoter region -100 bp away from ssTnI TSS. The MSP data show that the methylation level on CpG sites in -100 bp of ssTnI TSS is reduced in cells treated with 5-azacytidine. M, methylated; U, unmethylated. **c** Summary of the Bisulfite sequence data of methylation on CpG island in ssTnI promoter, indicating that there is no methylation change either in cells treated with 5-azacytidine or in control cells. **d** Summary of the Bisulfite sequence data of methylation on CpG dinucleotide region in ssTnI promoter area -100 bp away from the ssTnI TSS. The results show a significant decrease of DNA methylation on these regions in the cells treated with 5-azacytidine. *p < 0.05

5-azacytidine, a methylation inhibitor, was applied to 14-day-old myocardial cells to see whether ssTnI expression increases after the inhibition of ssTnI gene methylation. The expression of mRNA in myocardial cells that were prior treated with 5 μM 5-azacytidine for 24 h was significantly increased compared to that of the untreated control group (Fig. 3). The MSP data showed that 5-azacytidine could reduce DNA methylation in the CpG dinucleotides regions, while the methylation status of CpG island was not changed compared to the untreated control group (Fig. 4a and c). The BSP data in myocardial cells indicated that 5-azacytidine could reduce the methylation level of CpG dinucleotides region in ssTnI promoter compared to the untreated control group (Fig. 4b and d).

## Discussion

Troponin I is not only a structural protein but also an important regulatory protein that controls cardiac muscle contraction and relaxation. The ischemia and oxidative stress may cause cardiac muscle cell damages such as cell death, and then, the troponin protein is released from myocardial cells into blood. Therefore, troponin has become a critical myocardial marker to estimate the cardiac damages [26, 27]. Troponin I isoform switching during heart development was discovered almost three decades ago [28]. But the mechanisms of the fetal TnI (ssTnI) gene inactivation soon after birth are still unclear. The developmental switch from fetal to adult troponin I is found in lower and higher animals, and even in human beings. For this reason, TnI family has become a good model to investigate the regulation of cardiac proteins during heart development [4]. Re-expression of many fetal protein genes normally observed in fetal heart may occur in several pathological conditions such as cardiac hypertrophy and heart failure [29]. However no ssTnI re-expression is observed even in severe pathological conditions or in the heart at the end stage [9, 30]. We have cloned and characterized mouse ssTnI gene and our previous studies have showed that ssTnI gene was gradually shut down 15 days after birth in mouse hearts. Several important regulatory domains and elements on ssTnI promoters have been identified, such as SURE, a specific domain in upstream part of ssTnI gene, and one internal regulatory element (IRE) in the first intron region [4, 10–13]. We also predicted some transcription factors binding to the 100 bp upstream of ssTnI gene promoter, for example, specificity protein 1 (Sp1), myogenic differentiation (MyoD) and myocyte enhancer factor-2 (MEF-2). In addition, we have also found that the thyroid hormone could partially regulate the ssTnI gene expression in the developing mouse heart [9].

Recently, epigenetics, which is defined as the interaction of DNA methylation, histone modification and expression of noncoding RNAs, has been demonstrated to play an important role in the regulation of gene expression during the development [31–33]. Early studies have showed that methylation and demethylation play a role on regulation of gene expression during development, and there is an inverse correlation between DNA methylation and gene expression [33–35]. In mammalian genome, approximately 70 % of CpG are methylated [36], on the other hand, unmethylated CpG are grouped in clusters called “CpG island” in gene promoter regulatory regions. Interestingly, some methylation alterations do not occur in CpG island but in CpG dinucleotides (called CpG shores), and methylation on CpG island shores is strongly related to gene expression [18]. Our previous study has shown that histone modification, such as acetylation or methylation, may affect ssTnI expression during heart development [22]. However, little is known about the effect of DNA methylation on the regulation of ssTnI gene expression during heart development. In this study, our results have demonstrated that the DNA methylation on CpG dinucleotides regions of ssTnI promoter, located on 100 bp upstream from TSS, is gradually increased during heart development, but the level of DNA methylation on CpG island of ssTnI promoter does not change, suggesting the DNA methylation on CpG dinucleotides regions is associated with a down regulation of ssTnI gene expression in the heart. This CpG dinucleotides region is located in 100 bp upstream of the ssTnI TSS, which has been identified to be able to bind with several critical transcription factors, such as Sp1, MyoD and MEF-2. The enhanced methylation of this domain may inhibit the function of transcription factors and affect gene expression. Whereas the methylation of CpG island on ssTnI promoters is stable without any significant change at the different time points during the development, indicating that the CpG islands may not be the targets for regulation of ssTnI gene expression during heart development.

5′-azacytidine is a DNA methyl-transferase inhibitor that can reverse the DNA hypermethylation and reestablish gene expression [37, 38]. In this study, in order to confirm the methylation regulatory mechanism, 5-azacytidine has been used to treat myocardial cells to see whether ssTnI expression can be resumed by 5-azacytidine. Our results indicate that expression of ssTnI mRNA is increased in myocardial cells after treatment with 5-azacytidine (5 μM). The MSP and BSP data have shown that the increased DNA methylation in dinucleotides region located in -100 bp of ssTnI promoter is significantly reduced in myocardial cells treated with 5-azacytidine, while the CpG island was not influenced. Our study has demonstrated for the first time that methylation on the CpG dinucleotides region, not the CpG island, may play an important role in regulation of ssTnI gene expression during heart development.

## Conclusion

Our results indicate that DNA methylation occurs on CpG dinucleotides region, locating at -100 bp of ssTnI gene promoter, can cause the corresponding changes in ssTnI gene expression. But the methylation of CpG island on ssTnI gene promoter may not be critical in the regulation of ssTnI gene expression during heart development. In addition, DNA methylation inhibitor 5-azacytidine can increase ssTnI mRNA expression by reducing methylation on CpG dinucleotides region in ssTnI gene promoter. These data indicate that the epigenetic modification is involved in regulation of myofibril gene expression during heart development in mice.
